# High plasma renin activity associates with obesity-related diabetes and arterial hypertension, and predicts persistent hypertension after bariatric surgery

**DOI:** 10.1186/s12933-021-01310-w

**Published:** 2021-06-09

**Authors:** Lucia La Sala, Elena Tagliabue, Elaine Vieira, Antonio E Pontiroli, Franco Folli

**Affiliations:** 1 Lab. of Cardiovascular and Dysmetabolic Disease, IRCCS MultiMedica, 20138 Milan, Italy; 2 Value-based Healthcare Unit, IRCCS MultiMedica, Milan, Italy; 3 Postgraduate Program on Physical Education, Universidade Católica de Brasília, Taguatinga, DF 71966-700 Brazil; 4 Dipartimento di Scienze della Salute, Università degli Studi di Milano, 20142 Milan, Italy; 5 Unità di Endocrinologia, Ospedale San Paolo, ASST Santi Paolo e Carlo, Milan, Italy

**Keywords:** Renin, Aldosterone, Diabetes, HOMA index, Hypertension, Obesity, Weight loss, Bariatric surgery, Laparoscopic gastric banding (LAGB), Ferritin

## Abstract

**Background:**

Information about the renin–angiotensin–aldosterone system (RAAS) in obese individuals before and after bariatric surgery is scarce. Aim of this study was to analyze the RAAS in severely obese subjects, in relation to anthropometric and metabolic variables, with special reference to glucose tolerance.

**Methods:**

239 subjects were evaluated at baseline, and 181 one year after bariatric surgery [laparoscopic gastric banding (LAGB)].

**Results:**

At baseline, renin (plasma renin activity, PRA) was increased from normal to glucose tolerance and more in diabetes, also correlating with ferritin. After LAGB, the decrease of PRA and aldosterone was significant in hypertensive, but not in normotensive subjects, and correlatied with decrease of ferritin. PRA and glucose levels were predictive of persistent hypertension 1 year after LAGB.

**Conclusions:**

These data support the role of RAAS in the pathophysiology of glucose homeostasis, and in the regulation of blood pressure in obesity. Ferritin, as a proxy of subclinical inflammation, could be another factor contributing to the cross-talk between RAAS and glucose metabolism.

## Introduction

Arterial hypertension is a major risk factor for all cardiovascular diseases (CVD), including coronary heart disease (CHD), stroke, atrial fibrillation, heart failure (HF), aortic and peripheral arterial disease, and valvular heart disease [1]. Individuals with hypertension have a two- to threefold increased risk for all CVD events combined, as compared with non-hypertensive individuals [2]. The association between obesity and hypertension was shown prospectively in the Framingham Heart Study in the 1960s, when peripheral resistance was seen in obese hypertensive patients compared with normotensive obese patients [3].

Many factors are implicated in the link of central obesity with pathogenesis of hypertension, such as overactivation of the sympathetic nervous system (SNS) by insulin also present in obese patients [4], leptin, activation of the renin–angiotensin–aldosterone system (RAAS), sodium excretion, pressure natriuresis, and salt sensitivity [5, 6].

As the prevalence of obesity increases, also its co-morbidities [arterial hypertension, sleep apnea, impaired glucose tolerance (IGT) type 2 diabetes (T2D) with their associated cardiovascular (CV) risk], will increase as well. These co-morbidities are probably inter-related, due to hyperleptinemia and leptin resistance, oxidative stress, sub-clinical inflammation, endothelial dysfunction, sympathetic activation, insulin resistance (IR), and overactivation of RAAS [7]. In contrast, there are no data on the interplay between glucose tolerance and RAAS in obesity, in spite of the known cross-talk between insulin resistance and RAAS [8, 9].

Weight loss, obtained with diet alone, or in combination with pharmacological treatment, or bariatric surgery (BS), improves several metabolic variables and reduces arterial blood pressure [10, 11], preventing the appearance of T2D [12, 13] and of arterial hypertension [10]; in addition, BS, compared with medical treatment, improves quality of life and life expectancy [14, 15] and is cost-effective in the management of obesity [16, 17]. Most abnormalities improve after weight loss, and the evidences that these changes are related to each other and mainly with loss of abdominal fat are known [18]. Although previous data supported the benefits of laparoscopic gastric banding (LAGB) in greatly reducing risk factors for cardiovascular disease [19], the mechanisms through which weight reduction decreases the cardiovascular risk are not well established. There are some reports showing that weight loss, obtained through BS, reduces blood pressure, aldosterone (ALD) and renin plasma levels in hypertensive subjects, not in normotensive subjects [20]. It is well known that ALD affects glucose metabolism homeostasis and its high levels may increase the risk for diabetes mellitus (MESA, Multiethnic Study of Atherosclerosis) [21]. The pharmacological modulation of RAAS might be helpful in lowering the risk of appearance of diabetes and of hypertension [22, 23].

The first aim of this study was to analyze RAAS in obese subjects, candidate for BS, and to correlate RAAS with anthropometric and metabolic variables, especially with glucose tolerance. The second aim of this study was to analyze changes of basal components of RAAS system, of glucose metabolism, and of insulin sensitivity in obese subjects after bariatric surgery; the third aim was to develop predictive models of persistence of diabetes and of hypertension in spite of weight loss.

## Subjects and methods

239 obese subjects (43 men and 196 women, aged 40.6 ± 10.4 years, baseline BMI 44.5 ± 6.4 kg/m^2^), candidates for LAGB, were included in the study; all subjects gave written informed consent following the common protocol already described [24] approved by the local Ethics Committee, and were studied as in-patients under standardized conditions. All evaluations were performed at 30–45 days before intervention. Height, weight and waist circumference were measured in all subjects. Except for 6 patients with a pre-existing diagnosis of diabetes, diagnosis of was done by oral glucose tolerance test (OGTT) on fasting and/or 2-h plasma glucose [24]. Blood pressure was measured with an appropriate cuff three times in resting conditions in supine position, and the average of the second two readings was recorded. Hypertension was defined as systolic blood pressure (SBP) > 140 mmHg and/or diastolic pressure (DBP) > 90 mmHg [25] or current treatment with anti-hypertensive drugs (three patients). Supine (after 2 h in lying position) and upright (2 h after standing) plasma renin activity (PRA) and ALD, fasting plasma glucose (FPG), insulin, HbA1c, lipid profile, sodium and potassium electrolytes, ferritin, and creatinine were evaluated at 8.00 am under fasting conditions. The HOMA index (HOMA-IR) was also calculated [26]. Secondary hypertension was excluded on the basis of clinical and instrumental diagnosis; in particular, subjects with an elevated upright ALD/PRA ratio [27] underwent further investigation to rule out primary hyperaldosteronism, and were excluded from the study. Urinary cortisol levels were also assayed [28, 29]. All subjects underwent LAGB, and 181 of them could be analyzed 12 months after surgery. The post-surgery diet has already been described [24].

### Biochemical and hormonal assays

PRA and ALD were measured by radioimmunoassay (RIA) methods (DiaSorin, Vercelli, Italy, and MedicalSystem SpA, Genova, Italy, respectively). Serum electrolytes, blood glucose, insulin, creatinine, HbA1c, total and HDL cholesterol, triglycerides, leptin, ferritin, iron, and transferrin were evaluated as already indicated [24, 30, 31]. Urinary free cortisol levels were measured by immunologic chemiluminescent assay (Advia Centaur Bayer, BAYER- Diagnostics). All measurements were carried out in the same laboratory; intra- and inter-assay coefficients of variation (CVs) for insulin, PRA, and ALD assay were 3.0–5.0%, 3.6–6.1%, and 3.3–5.6%, respectively.

### Statistical analysis

Data are presented as mean ± SD or as frequencies. Subjects were divided into normal glucose tolerance (NGT), impaired glucose tolerance (IGT) and T2D. Intrasubject changes between baseline and after 12 months from LAGB were analyzed by paired t test or non-parametric Wilcoxon signed rank test, as appropriate. Since a normal distribution of data was not assured, data were compared in the glucose tolerance groups by non-parametric Kruskal–Wallis test followed by Bonferroni adjustment for multiple comparisons. Normotensive and hypertensive were compared by non-parametric Wilcoxon test. Correlation between baseline PRA levels and other collected variables was evaluated. Multivariable regression analysis was performed for some outcomes, with models including significant variables at univariate analysis.

### Modeling

Logistic modeling for the persistence of diabetes after LAGB was calculated by taking into account variables significantly associated with diabetes at 1 year. Logistic regression models for the association between hypertension at 12 months after LAGB and PRA, adjusted for glycemic parameters or groups were implemented and correspondent receiver operating characteristic (ROC) curves, with AUC (area under the ROC curve), were drawn. Based on the logistic model, a nomogram was built to predict the probability to have persistence of arterial hypertension at 1 year after BS, in spite of weight loss. *p* less than 0.05 were considered statistically significant; for multiple comparisons, a *p* value < 0.0167 was considered statistically significant. All statistical analyses were performed with SAS Software v 9.4 (SAS Institute Inc., Cary, NC, USA) or STATA12 for Macintosh (Stata Corporation, College Station, TX).

## Results

### Baseline conditions

At baseline NGT subjects were younger than IGT and T2DM subjects, without differences in sex proportion and BMI among groups. Triglycerides were different among the three groups; in addition, diastolic BP, ferritin were higher for T2D than IGT and NGT, and HDL-cholesterol were lower; the frequency of hypertensive subjects was higher for IGT and T2D than NGT. Transferrin, iron, and albumin did not differ among the three groups. There was a trend towards increased supine PRA between NGT, IGT and T2D, although not significant (Table 1).

**Table 1:** Baseline details of all subjects in the study divided by glucose tolerance

	NGT	IGT	T2D	Overall *p*^^^	NGT vs. IGT*	NGT vs. T2D*	IGT vs. T2D*
N (M/W)	139 (26/113)	66 (10/56)	34 (7/27)	0.754			
Age (yr)	37.5 ± 9.6	43.2 ± 10.0	46.2 ± 8.3	< 0.0001	0.0002	< 0.0001	0.2313
Weight (kg)	118.4 ± 20.3	119.4 ± 20.3	123.0 ± 21.4	0.4000	0.7711	0.1883	0.2709
BMI (kg/m^2^)	44.0 ± 5.8	44.2 ± 5.7	45.6 ± 5.7	0.1331	0.1847	0.0746	0.4498
Waist circum (cm)	120.6 ± 14.3	121.8 ± 12.4	129.5 ± 12.9	0.0029	0.3278	0.0011	0.0061
FPG (mg/dl)	97.3 ± 12.8	107.6 ± 18.1	165.5 ± 70.6	< 0.0001	0.0004	< 0.0001	< 0.0001
1hPG (mg/dl)	155.4 ± 44.2	201.0 ± 47.8	306.8 ± 94.7	< 0.0001	< 0.0001	< 0.0001	< 0.0001
2hPG (mg/dl)	107.7 ± 20.9	163.4 ± 16.1	286.0 ± 82.1	< 0.0001	< 0.0001	< 0.0001	< 0.0001
Insulin (µU/ml)	18.9 ± 11.8	18.1 ± 9.0	23.5 ± 16.9	0.1374	0.8736	0.0542	0.0844
HOMA-IR	4.6 ± 3.4	4.9 ± 2.9	9.2 ± 6.2	< 0.0001	0.2888	< 0.0001	< 0.0001
HbA1c (%)	5.8 ± 0.6	6.2 ± 0.7	7.8 ± 1.8	< 0.0001	0.0006	< 0.0001	< 0.0001
Total cholesterol (mg/dl)	201.3 ± 40.2	206.8 ± 40.2	215.1 ± 50.0	0.2396	0.2995	0.1225	0.4683
HDL-cholesterol (mg/dl)	48.5 ± 12.0	50.6 ± 18.0	41.8 ± 10.6	0.0126	0.9916	0.0051	0.0067
Triglycerides (mg/dl)	131.5 ± 72.1	144.9 ± 67.5	219.3 ± 115.6	< 0.0001	0.0418	< 0.0001	0.0010
Systolic BP (mmHg)	131.5 ± 15.4	131.4 ± 13.3	138.1 ± 17.6	0.1066	0.8417	0.0395	0.0761
Diastolic BP (mmHg)	82.4 ± 8.4	82.8 ± 9.4	87.8 ± 10.9	0.0113	0.8700	0.0032	0.0140
Hypertension (Y/N)	29/110	24/42	17/17	0.0019	0.0258	0.0011	0.4431
Creatinine (mg/dl)	0.7 ± 0.1	0.7 ± 0.1	0.7 ± 0.2	0.4839	0.2285	0.9787	0.4878
Leptin (ng/ml)	37.7 ± 18.4	41.3 ± 18.2	43.2 ± 37.1	0.6192	0.3873	0.7705	0.5264
Ferritin (ng/ml)	86.1 ± 92.1	83.6 ± 93.6	154.3 ± 178.2	0.0919	0.8618	0.0435	0.0362
Transferrin (mg/ml)	2.7 ± 0.45	2.7 ± 0.53	2.8 ± 0.58	0.8545	0.7821	0.5779	0.7808
Iron (µg/dl)	84.2 ± 33.15	78.9 ± 28.59	81.4 ± 26.65	0.5331	0.8963	0.3110	0.5068
Albumin (g/l)	58.2 ± 4.44	58.0 ± 3.63	56.6 ± 5.44	0.1826	0.8187	0.0855	0.1274
Calcium (mmol/l)	2.3 ± 0.1	2.3 ± 0.1	2.3 ± 0.1	0.2797	0.1082	0.6496	0.4999
Sodium (mmol/l)	140.6 ± 2.3	140.6 ± 2.3	140.3 ± 2.9	0.7137	0.8625	0.4656	0.4203
Supine PRA (ng/ml/h)	1.8 ± 2.3	2.2 ± 4.2	4.3 ± 8.0	0.2543	0.2775	0.3262	0.1229
Upright PRA (ng/ml/h)	3.2 ± 3.9	3.5 ± 5.2	7.5 ± 13.5	0.4492	0.2339	0.9617	0.3440
Supine ALD (ng/dl)	3.6 ± 2.7	3.1 ± 2.7	3.4 ± 1.9	0.1871	0.0932	0.8555	0.1464
Upright ALD (ng/dl)	8.4 ± 4.7	7.2 ± 5.0	9.7 ± 7.4	0.0739	0.0289	0.8127	0.1123
Upright ALD/PRA	5.0 ± 6.5	5.8 ± 9.2	5.2 ± 7.0	0.9172	0.9399	0.6749	0.7665
Urinary cortisol (ng/24 h)	117.6 ± 46.9	115.2 ± 42.1	110.2 ± 51.4	0.5540	0.7934	0.2833	0.4302
Uric acid (mg/dl)	5.1 ± 1.2	5.0 ± 1.1	5.4 ± 1.4	0.5677	0.5804	0.4695	0.2901
Hypoglycemic therapy^a^	0.0 ± 0.0	0.0 ± 0.0	1.5 ± 3.3	< 0.0001	1.0000	< 0.0001	< 0.0001

*NGT *normal glucose tolerance, *IGT *impaired glucose tolerance, *T2D *type 2 diabetes, *BMI *body mass index, *FPG *fasting plasma glucose, *1hPG *1-hour plasma glucose, *2hPG *2-hour plasma glucose, *BP *blood pressure, *PRA *plasma renin activity, *ALD *aldosterone

^^^Non-parametric Kruskal–Wallis test

*Multiple comparisons are based on pairwise Wilcoxon test with Bonferroni adjustment (*p* < 0.0167 is considered statistically significant)

^a^Pills per day

Table 2 shows that hypertensive subjects (n = 70) were older, with higher BP and supine PRA than normotensive subjects (n = 169). In addition, for waist circumference, total cholesterol, triglycerides, ferritin, uric acid and glycemic parameters except that insulin, differences were significant between normotensive and hypertensive subjects. Baseline supine PRA levels were significantly correlated with age, glycemic parameters, leptin, ferritin, calcium, sodium, ALD and uric acid (Table 3). Reverse associations were also calculated; HOMA-IR, FPG and 1hPPg and 2hPPG correlated with ferritin. At multivariate analysis, glucose tolerance, blood glucose, HOMA-IR, and ferritin were predictors of supine and upright PRA; age and supine PRA were predictors of supine ALD, and age, upright PRA and blood glucose were predictors of upright ALD (results not shown).

**Table 2:** Baseline details of all subjects in the study divided by hypertension

	Normotensive	Hypertensive	p*
N (M/W)	169	70	
Age (yr)	38.2 ± 9.7	46.4 ± 9.7	< 0.0001
Weight (kg)	117.7 ± 19.2	122.7 ± 21.7	0.1454
BMI (kg/m^2^)	44.0 ± 6.2	45.8 ± 6.8	0.0687
Waist circumference (cm)	120.3 ± 13.5	127.0 ± 13.7	0.0005
FPG (mg/dl)	103.1 ± 23.9	126.6 ± 55.8	< 0.0001
1hPG (mg/dl)	175.1 ± 55.8	222.4 ± 99.7	0.0006
2hPG (mg/dl)	136.3 ± 52.8	177.6 ± 93.4	0.0004
Insulin (µU/ml)	19.3 ± 13.2	19.0 ± 9.0	0.3967
HOMA-IR	5.0 ± 4.0	6.1 ± 4.3	0.0240
HbA1c (%)	6.0 ± 0.9	6.7 ± 1.4	< 0.0001
Glucose tolerance (NGT/IGT/T2D)	110/42/17	29/24/17	0.0019
Total cholesterol (mg/dl)	199 ± 40.5	215.5 ± 42.1	0.0025
HDL-cholesterol (mg/dl)	48.3 ± 14.3	47.6 ± 13.2	0.7593
Triglycerides (mg/dl)	137.7 ± 72.2	174.0 ± 100.5	0.0046
Systolic BP (mmHg)	126.2 ± 9.0	143.3 ± 17.9	< 0.0001
Diastolic BP (mmHg)	79.7 ± 6.6	89.5 ± 10.0	< 0.0001
Creatinine (mg/dl)	0.7 ± 0.1	0.7 ± 0.2	0.1204
Leptin (ng/ml)	40.1 ± 19.4	38.6 ± 21.9	0.4670
Ferritin (ng/ml)	76.5 ± 83.6	135.6 ± 140.6	0.0004
Transferrin (mg/ml)	2.8 ± 0.5	2.6 ± 0.5	0.0637
Iron (µg/dl)	82.1 ± 33.7	82.8 ± 25.3	0.8775
Albumin (g/l)	57.7 ± 4.23	58.3 ± 4.7	0.3213
Calcium (mmol/l)	2.3 ± 0.1	2.4 ± 0.1	0.1770
Sodium (mmol/l)	140.6 ± 2.5	140.3 ± 2.3	0.5040
Supine PRA (ng/ml/h)	1.7 ± 3.0	4.1 ± 6.9	0.0159
Upright PRA (ng/ml/h)	3.0 ± 4.1	6.9 ± 10.9	0.1200
Supine ALD (ng/dl)	3.3 ± 2.2	3.5 ± 3.1	0.6808
Upright ALD (ng/dl)	7.9 ± 4.5	9.0 ± 6.5	0.4968
Upright ALD/PRA	4.8 ± 5.1	5.9 ± 10.6	0.0644
Urinary cortisol (ng/24 h)	113.8 ± 45.8	115.1 ± 47.3	0.8875
Uric acid (mg/dl)	5.0 ± 1.2	5.5 ± 1.2	0.0022
Hypoglycemic therapy^a^	0.2 ± 1.4	0.4 ± 1.4	0.0176

*BMI *body mass index, *FPG *fasting plasma glucose, *1hPG *1-hour plasma glucose, *2hPG* 2-hour plasma glucose, *NGT *normal glucose tolerance, *IGT *impaired glucose tolerance, *T2D *type 2 diabetes, *BP *blood pressure, *PRA *plasma renin activity, *ALD *aldosterone

*Non-parametric Wilcoxon test

^a^Pills per day

**Table 3:** Correlations between baseline characteristics and supine renin activity

	N	Rho	*p*
Age (yr)	225	0.14707	0.0274
FPG (mg/dl)	219	0.39719	< 0.0001
1hPG (mg/dl)	217	0.32632	< 0.0001
2hPG (mg/dl)	216	0.29338	< 0.0001
HOMA-IR	217	0.20082	0.0032
HbA1c (%)	217	0.37418	< 0.0001
Leptin (ng/ml)	50	0.29842	0.0353
Ferritin (ng/ml)	218	0.25217	0.0004
Calcium (mmol/L)	192	0.21115	0.0033
Sodium (mmol/l)	225	− 0.21633	0.0011
Upright PRA (ng/ml/h)	224	0.89527	< 0.0001
Supine ALD (ng/dl)	216	0.23005	0.0007
Upright ALD (ng/dl)	216	0.26412	< 0.0001
Upright ALD/PRA	215	− 0.23321	0.0006
Uric acid (mg/dl)	218	0.17363	0.0102
Hypoglycemic therapy^a^	190	0.19768	0.0063

Only significant correlations are shown

*FPG *fasting plasma glucose, *1hPG *1-hour plasma glucose, *2hPG *2-hour plasma glucose, *PRA *plasma renin activity, *ALD *aldosterone

^a^Pills per day

### Effects of surgery

**Fig. 1:**
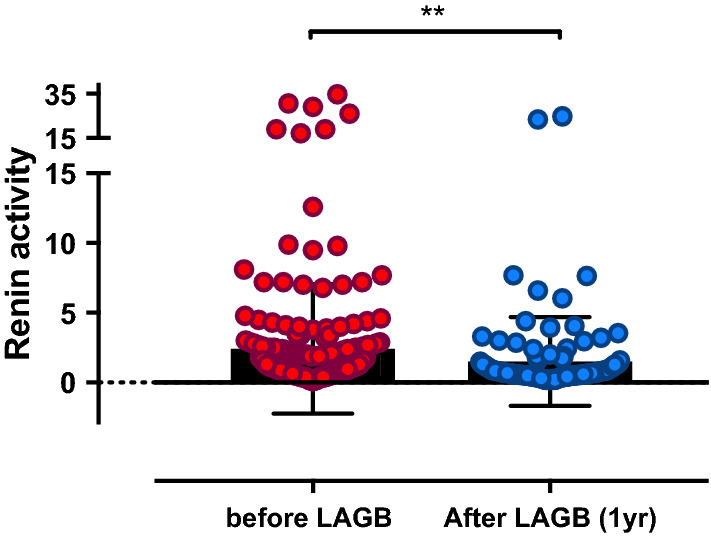
Comparison between levels of supine plasmatic renin activity (PRA) before and post-laparoscopic adjustable gastric banding (LAGB) in obese (n = 110) people. Non-parametric Wilcoxon signed rank test for paired data was used to test significance. **p = 0.0035

In subjects re-evaluated one year after BS, all changes observed were as expected. Subjects re-evaluated after one year differed from subjects lost to follow-up only for age (41.96 ± 10.13 vs. 36.30 ± 9.96 year, p = 0.0002), not for any other variable (data not shown). Weight, BMI, waist circumference, glycemic parameters, triglycerides and BP significantly decreased after LAGB (Table 4). Also supine PRA (Table 4; Fig. 1), supine ALD and ferritin significantly decreased after bariatric BS. Particularly, weight, BMI, waist circumference and glycemic parameters decreased in a similar manner in NGT, IGT, and T2DM subjects (Table 5). Triglycerides, HDL and BP significantly improve after LAGB in all groups, except for SBP and DBP that did not change in IGT subjects. Generally, NGT was the group in which changes after BS were most evident, including hypertension. Metabolic and glycemic parameters significantly decreased after LAGB in both normotensive and hypertensive subjects (Table 6). Also HDL, triglycerides, BP and ferritin improved after intervention, but transferrin, iron and albumin did not vary. Particularly, hypertensive subjects were those with the greatest changes. Supine PRA and ALD only significantly decreased in hypertensive subjects, not in normotensive subjects. Both glucose tolerance and frequency of hypertension improved after surgery, but a few patients required de-novo treatment of hypertension. At multivariable analysis, decrease of supine PRA and of upright PRA was predicted by decrease of ferritin; decrease of both supine and upright ALD was predicted by decrease of waist circumference (data not shown).

**Table 4:** Differences between baseline values and 12 months after bariatric surgery

	Baseline		12 months		*p*^^^
	N	Mean (± SD)	N	Mean (± SD)	
Weight (kg)	239	119.2 ± 19.97	181	97.3 ± 16.61	< 0.0001
BMI (kg/m^2^)	239	44.4 ± 5.79	181	36.4 ± 5.21	< 0.0001
Waist circumference (cm)	238	122.2 ± 13.84	174	107.1 ± 13.5	< 0.0001
FPG (mg/dl)	233	110.4 ± 38.05	171	95.8 ± 17.33	< 0.0001
1hPG (mg/dl)	226	189.8 ± 75.31	176	158.2 ± 58.57	< 0.0001
2hPG (mg/dl)	225	149.1 ± 70.52	169	118.7 ± 47.94	< 0.0001*
Insulin (µU/ml)	230	19.4 ± 11.25	160	10.0 ± 5.21	< 0.0001
HOMA-IR	230	5.4 ± 4.08	147	2.4 ± 1.5	< 0.0001
HbA1c (%)	229	6.2 ± 1.1	155	5.7 ± 0.7	< 0.0001
Total cholesterol (mg/dl)	239	203.9 ± 41.51	181	204.7 ± 42.54	0.4949*
HDL-cholesterol (mg/dl)	225	48.1 ± 13.93	168	53.4 ± 12.48	< 0.0001*
Triglycerides (mg/dl)	239	148.8 ± 83.08	171	107.4 ± 56.29	< 0.0001
Systolic BP (mmHg)	194	132.3 ± 15.21	136	127.1 ± 12.9	0.0001
Diastolic BP (mmHg)	194	83.2 ± 9.24	136	80.3 ± 8.89	0.0004
Creatinine (mg/dl)	239	0.7 ± 0.15	167	0.7 ± 0.07	0.2882
Leptin (ng/ml)	52	39.5 ± 20.22	52	20.6 ± 12.4	< 0.0001*
Ferritin (ng/ml)	232	95.5 ± 100.98	181	83.3 ± 84.81	0.0164
Transferrin (mg/ml)	221	2.7 ± 0.49	155	2.6 ± 0.48	0.3279
Iron (µg/dl)	235	82.3 ± 31.08	181	85.8 ± 30.43	0.2195
Albumin (g/l)	232	57.9 ± 4.40	159	58.6 ± 4.38	0.8788
Calcium (mmol/l)	202	2.3 ± 0.11	163	2.4 ± 0.11	0.0055*
Sodium (mmol/l)	239	140.5 ± 2.42	181	141.0 ± 2.44	0.0014*
Supine PRA (ng/ml/h)	225	2.4 ± 4.63	115	1.3 ± 2.50	0.0035
Upright PRA (ng/ml/h)	224	4.1 ± 7.08	116	3.1 ± 6.09	0.1821
Supine ALD (ng/dl)	230	3.3 ± 2.53	113	2.9 ± 3.7	0.0005
Upright ALD (ng/dl)	230	8.2 ± 5.25	114	8.3 ± 8.2	0.0439
Upright ALD/PRA	215	5.2 ± 7.23	111	5.7 ± 6.9	0.8917
Urinary cortisol (ng/24 h)	180	114.1 ± 46.06	96	106.8 ± 45.5	0.0170*
Uric acid (mg/dl)	232	5.2 ± 1.20	181	4.7 ± 1.27	< 0.0001*
Hypoglycemic therapy^a^	239	0.2 ± 1.4	157	0.3 ± 2.2	1.0000

*BMI *body mass index, *FPG *fasting plasma glucose, *1hPG *1-hour plasma glucose, *2hPG * 2-hour plasma glucose, *BP *blood pressure, *PRA *plasma renin activity, *ALD *aldosterone

^^^Non-parametric Wilcoxon Signed Rank test for paired data

*Paired T test

^a^Pills per day

**Table 5:** Changes induced by weight loss according to glucose tolerance

	All	*p*^^^	NGT	*p*^^^	IGT	*p*^^^	T2DM	*p*^^^
Δ Weight	− 21.7 ± 13.0	< 0.0001	− 22.2 ± 11.9	< 0.0001	− 21.4 ± 15.6	< 0.0001	− 18.6 ± 10.6	< 0.0001
Δ BMI	− 8.1 ± 4.5	< 0.0001	− 8.0 ± 4.1	< 0.0001	− 8.2 ± 5.4	< 0.0001	− 7.9 ± 4.1	< 0.0001
Δ WC	− 15.4 ± 10.1	< 0.0001	− 16.5 ± 9.5	< 0.0001	− 13.6 ± 11.8	< 0.0001	− 13.2 ± 8.3	< 0.0001
Δ FPG	− 15.6 ± 25.8	< 0.0001	− 8.7 ± 15.8	< 0.0001	− 13.7 ± 17.5	< 0.0001	− 45.5 ± 45.5	< 0.0001
Δ 1hPG	− 36.7 ± 48.8	< 0.0001	− 23.4 ± 45.8	< 0.0001	− 48.4 ± 47.2	< 0.0001	− 62.5 ± 46.5	< 0.0001
Δ 2hPG	− 34.7 ± 53.9	< 0.0001*	− 6.2 ± 34.2	0.1018*	− 46.5 ± 40.4	< 0.0001*	− 113.5 ± 53.7	< 0.0001*
Δ Insulin	− 8.6 ± 9.7	< 0.0001	− 9.5 ± 11.2	< 0.0001	− 6.9 ± 7.2	< 0.0001	− 9.1 ± 8.2	< 0.0001
Δ HOMA-IR	− 2.8 ± 3.3	< 0.0001	− 2.6 ± 3.2	< 0.0001	− 2.3 ± 2.4	< 0.0001	− 4.8 ± 4.2	< 0.0001
Δ HbA1c	− 0.6 ± 1.0	< 0.0001	− 0.3 ± 0.6	< 0.0001	− 0.5 ± 0.7	< 0.0001	− 1.7 ± 1.7	< 0.0001
Δ Total chol	1.7 ± 30.7	0.4949*	0.0 ± 29.8	0.9971*	− 0.2 ± 31.7	0.9711*	10.2 ± 30.1	0.1458*
Δ HDL-chol	6.3 ± 9.8	< 0.0001*	6.9 ± 9.7	< 0.0001*	5.4 ± 9.7	0.0006*	5.2 ± 9.9	0.0386*
Δ Triglycerides	− 41.2 ± 68.9	< 0.0001	− 34.6 ± 59.6	< 0.0001	− 35.2 ± 54.5	< 0.0001	− 76.5 ± 104.9	0.0019
Δ Systolic BP	− 5.2 ± 14.7	0.0001	− 6.1 ± 15.5	0.0007	− 1.4 ± 11.4	0.5372	− 11.6 ± 17.4	0.0212
Δ Diastolic BP	− 3.2 ± 11.2	0.0014	− 3.8 ± 10.8	0.0052	0.0 ± 10.6	0.9073	− 8.8 ± 12.6	0.0114
Δ Creatinine	0.0 ± 0.1	0.2882	0.0 ± 0.1	0.9522	0.0 ± 0.1	0.2774	0.0 ± 0.1	1.0000
Δ Leptin	− 17.7 ± 12.8	< 0.0001*	− 17.1 ± 9.4	< 0.0001*	− 21.1 ± 16.4	< 0.0001*	− 6.3 ± 4.8	0.0781*
Δ Ferritin	− 16.2 ± 76.6	0.0164	− 18 ± 44.7	0.0095	− 7.4 ± 43.3	0.5239	− 31.1 ± 190.6	1.0000
Δ Transferrin	− 0.0 ± 0.4	0.3279	− 0.0 ± 0.4	0.7014	− 0.0 ± 0.5	0.5159	− 0.1 ± 0.4	0.4132
Δ Iron	1.8 ± 28.7	0.4731	2.2 ± 30.0	0.5303	3.6 ± 27.2	0.3900	− 3.7 ± 27.8	0.5529
Δ Albumin	− 0.1 ± 3.8	0.8788	0.3 ± 3.8	0.4367	− 0.5 ± 4.2	0.4401	− 0.6 ± 2.9	0.3637
Δ Calcium	0.0 ± 0.1	0.0055*	0.0 ± 0.1	0.0099*	0.0 ± 0.1	0.1731*	0.0 ± 0.1	0.8552*
Δ Sodium	0.8 ± 3.0	0.0014*	0.5 ± 3.2	0.1291*	1.1 ± 2.4	0.0039*	1.5 ± 2.9	0.0321*
Δ Supine PRA	− 0.9 ± 4.5	0.0035	− 0.4 ± 3.8	0.0067	− 1.1 ± 4.7	0.2947	− 3.1 ± 7.0	0.5195
Δ Upright PRA	− 1.0 ± 7.9	0.1821	0.0 ± 7.2	0.5256	− 1.5 ± 5.5	0.2745	− 5.0 ± 14.5	0.5186
Δ Supine ALD	− 0.6 ± 4.3	0.0005	− 0.7 ± 5.3	0.0016	− 0.4 ± 2.0	0.2624	− 1.0 ± 2.8	0.2439
Δ Upright ALD	− 0.4 ± 8.7	0.0439	0.0 ± 10.3	0.1674	− 0.1 ± 4.5	0.5056	− 3.1 ± 8.1	0.1099
Δ Upright A/PRA	− 0.5 ± 8.7	0.8917	− 0.1 ± 9.3	0.8980	− 0.5 ± 7.0	0.2851	− 2.9 ± 9.9	0.3804
Δ Urinary cortisol	− 15.9 ± 56.3	0.0170*	− 21.5 ± 49.9	0.0060*	− 9.7 ± 68.8	0.5379*	− 3.1 ± 58.8	0.8714*
Δ Uric acid	− 0.4 ± 1.1	< 0.0001*	− 0.5 ± 1.1	< 0.0001*	− 0.4 ± 1.1	0.0138*	− 0.2 ± 1.5	0.5167*
Δ Hypoglycemic^a^	0.0 ± 1.2	1.0000	0.0 ± 0.0	N.E	0.1 ± 0.5	1.0000	− 0.2 ± 3.4	1.0000

−  indicates decrease

*NGT *normal glucose tolerance, *IGT *impaired glucose tolerance, *T2D *type 2 diabetes, *BMI *body mass index, *WC *waist circumference, *FPG *fasting plasma glucose, *1hPG *1-hour plasma glucose, *2hPG *2-hour plasma glucose, *BP *blood pressure, *PRA *plasma renin activity, *ALD *aldosterone, *ALD/PRA* Upright A/PRA, *N.E *not estimable

^Non-parametric Wilcoxon signed rank test for paired data

*Paired T test

^a^Pills per day

**Table 6:** Changes induced by weight loss according to hypertension

	NT	*p*^^^	HT	*p*^^^
Δ Weight	− 22.0 ± 12.2	< 0.0001	− 20.9 ± 14.6	< 0.0001
Δ BMI	− 8.2 ± 4.3	< 0.0001	− 7.9 ± 5.0	< 0.0001
Δ Waist circumference	− 15.4 ± 10.0	< 0.0001	− 15.3 ± 10.3	< 0.0001
Δ FPG	− 11.5 ± 18.2	< 0.0001	− 24.1 ± 35.7	< 0.0001
Δ 1hPG	− 34.3 ± 45.3	< 0.0001	− 41.5 ± 55.6	< 0.0001
Δ 2hPG	− 28.1 ± 45.9	< 0.0001*	− 48.1 ± 65.8	< 0.0001*
Δ Insulin	− 8.8 ± 10.6	< 0.0001	− 8.2 ± 7.6	< 0.0001
Δ HOMA-IR	− 2.5 ± 3.1	< 0.0001	− 3.3 ± 3.5	< 0.0001
Δ HbA1c	− 0.5 ± 0.9	< 0.0001	− 0.9 ± 1.1	< 0.0001
Δ Total cholesterol	0.5 ± 29.5	0.8489*	4.0 ± 33.2	0.3935*
Δ HDL-cholesterol	6.2 ± 9.7	< 0.0001*	6.7 ± 10.1	< 0.0001*
Δ Triglycerides	− 38.8 ± 65.0	< 0.0001	− 46.4 ± 76.9	< 0.0001
Δ Systolic BP	− 2.8 ± 11.4	0.0156	− 9.0 ± 18.4	0.0003
Δ Diastolic BP	− 1.4 ± 9.7	0.0948	− 5.8 ± 12.2	0.0004
Δ Creatinine	0.0 ± 0.1	0.7750	0.0 ± 0.1	0.1481
Δ Leptin	− 19.1 ± 12.9	< 0.0001	− 15.5 ± 12.6	0.0002
Δ Ferritin	− 15.9 ± 41.8	0.0042	− 16.8 ± 125.5	0.8130
Δ Transferrin	0.0 ± 0.4	0.5693	0.1 ± 0.4	0.3586
Δ Iron	1.7 ± 28.8	0.5846	1.9 ± 28.9	0.6453
Δ Albumin	0.2 ± 4.2	0.6064	− 0.2 ± 3.7	0.6609
Δ Calcium	0.04 ± 0.12	0.0037*	0.02 ± 0.11	0.1442*
Δ Sodium	0.41 ± 0.30	0.2717*	1.4 ± 0.36	< 0.0001*
Δ Supine PRA	0.1 ± 3.0	0.3616	− 2.7 ± 6.0	0.0005
Δ Upright PRA	0.4 ± 6.0	0.8130	− 3.6 ± 10.0	0.0605
Δ Supine ALD	− 0.2 ± 4.8	0.0529	− 1.4 ± 3.2	0.0015
Δ Upright ALD	0.3 ± 10.0	0.2545	− 1.6 ± 5.5	0.0796
Δ Upright ALD/PRA	− 0.5 ± 8.2	0.7108	− 0.7 ± 9.6	0.5834
Δ Urinary free cortisol	− 13.4 ± 56.5	0.1085*	− 20.4 ± 56.7	0.0731*
Δ Uric-acid	− 0.4 ± 1.2	0.0009*	− 0.5 ± 1.0	0.0011*
Δ Hypoglycemic therapy^a^	0.1 ± 1.2	0.7500	− 0.1 ± 1.2	1.0000

– indicates decrease

*NT *normotensive, *HT *hypertensive, *BMI *body mass index, *FPG *fasting plasma glucose, *1hPG *1-hour plasma glucose, *2hPG *2-hour plasma glucose, *BP *blood pressure, *PRA *plasma renin activity, *ALD *aldosterone

^^^Non-parametric Wilcoxon signed rank test for paired data

*Paired T test

^a^Pills per day

### Modelling

**Fig. 2:**
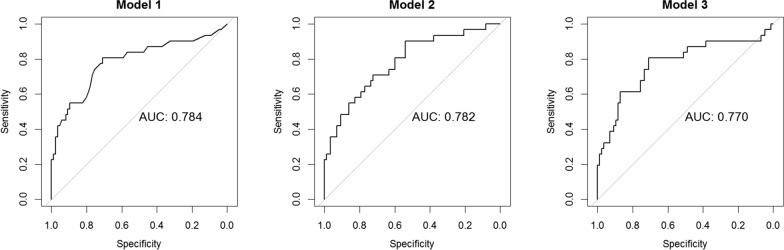
Receiver operating characteristic (ROC) curves and AUC (area under the ROC curve) of logistic regression models for the probability of hypertension at 12 months after laparoscopic adjustable gastric banding (LAGB)

**Fig. 3:**
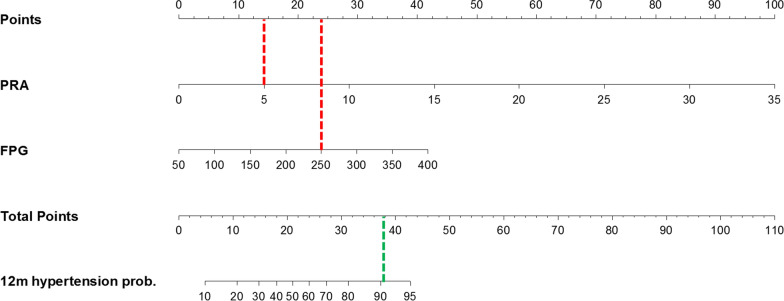
Nomogram for the prediction of the probability (prob.) of hypertension at 12 months after laparoscopic adjustable gastric banding (LAGB), based on plasma renin activity (PRA) and fasting blood glucose (FPG). For each patient variable (PRA and FPG), a vertical line is drawn from the value on the bar for that variable to the upper scale of points (dotted red lines show that PRA = 5 corresponds to 14 points in the upper bar and FPG = 250 corresponds to 24 points). The sum of these two points is then located on the scale indicating the “Total Points” (here: 14 + 24 = 38 total points), and a vertical line is drawn downwards (green dotted line). Where this line intersects, the scale for 12 m hypertension probability gives the percentage risk of hypertension at 12 months after LAGB. Values outside the indicated bar should be read as risk < 10% (for Total Points < 5) or risk > 95% (for Total Points > 42), respectively. In the example above, a subject with PRA of 5 and FPG of 250 has about 90% risk of hypertension at 12 months despite weight loss

The only predictor of diabetes 1 year after BS was represented by fasting blood glucose (or 1hPPG or 2hPPG) or glucose tolerance at baseline. Of the patients with hypertension at 1 year after LAGB, 29 were hypertensive at baseline and 2 became hypertensive during the follow-up. Association between hypertension at 12 months from LAGB and baseline supine PRA was evaluated by three logistic regression models in which supine PRA was adjusted either by glucose tolerance groups, FPG and 2hPG (Model 1, Model 2 and Model 3, respectively—Table 7). Increase in supine PRA levels was associated with probability of hypertension at 12 months, which ranged from 47 to 63%, for each ng/ml/h of PRA increase. In Model 1, IGT subjects had 3.60 times probability to be hypertensive at 12 months after surgery as compared with NGT. That probability increased for T2D if compared with NGT (OR: 4.85; 95% CI 1.15–20.56). Model 2 and 3 showed that for each mg/dl increase of FPG or 2hPG, the probability of hypertension at 12 months after LAGB increased of 2 and 1%, respectively. Figure 2 shows ROC curves and AUC for each logistic model. The nomogram based on Model 2 is a useful tool to predicted probability hypertension at 12 months from BS basing on subject characteristics. For instance, a subject with supine PRA = 5.0 ng/ml/h and FPG = 250 mg/dl has about 90% risk of hypertension at 1 year, despite weight loss (Fig. 3).

**Table 7:** Logistic models for the probability of hypertension 12 months after LAGB

Variables	Model 1		Model 2		Model 3	
	OR (95% CI)	*p*	OR (95% CI)	*p*	OR (95% CI)	*p*
Supine PRA (ng/ml/h)	1.63 (1.19–2.24)	0.0023	1.47 (1.10–1.96)	0.0085	1.52 (1.14–2.03)	0.0049
Glucose tolerance groups						
NGT	1.00 (Reference)		–	–	–	–
IGT	3.60 (1.22–10.60)	0.0319	–	–	–	–
T2D	4.85 (1.15–20.56)	0.0201	–	–	–	–
FPG	–	–	1.02 (1.00–1.03)	0.0453	–	–
2hPG	–	–	–	–	1.01 (1.00–1.02)	0.0470
	AUC = 0.784		AUC = 0.782		AUC = 0.770	

Model 1 is based on supine PRA and glucose tolerance groups. Model 2 is based on supine PRA and FPG. Model 3 is based on supine PRA and 2hPG

*OR *odds ratio, *CI *confidence interval, *PRA *plasma renin activity, *NGT *normal glucose tolerance, *IGT *impaired glucose tolerance, *T2D *Type 2 diabetes, *FPG *fasting plasma glucose, *2hPG *2-hour plasma glucose, *AUC *area under the curve

## Discussion

In this study, in obese subjects undergoing bariatric surgery, supine PRA was higher in hypertensive than in normotensive subjects, and progressively higher in normal glucose tolerance, impaired glucose tolerance, and diabetes. Before BS, supine PRA correlated with HOMA-IR, and both PRA and HOMA-IR correlated with ferritin. Interestingly, decrease of supine PRA at 12 months after LAGB only occurred in hypertensive subjects, as previously reported, with no difference among groups of glucose tolerance. Also, decrease of supine PRA correlated with decrease of HOMA-IR and with decrease of ferritin, suggesting that changes of ferritin are pivotal to change of HOMA-IR and of supine PRA. Similar, albeit less significant, was the behavior of upright PRA, and of both supine and upright aldosterone. Obese individuals frequently develop hypertension, which is largely attributable to RAAS overactivity [6]. Logistic modeling demonstrated that glucose tolerance (or FPG or 2hPPG) and PRA are predictors of persistent hypertension at 1 year after BS; a nomogram was derived with probabilities of persistent hypertension based on FPG and PRA at baseline. These results also point to a contribution of ferritin in the interplay between the RAAS and insulin sensitivity. However, there are other aspects that deserve consideration in this possible interplay. For instance, BS is also accompanied by reduced sub-clinical inflammation (not evaluated in this study); studies have shown decrease of inflammatory markers after BS [32], and ferritin is considered also a marker of inflammation [33]. In this study, changes in ferritin levels among NGT, IGT, and DM were not accompanied by changes in transferrin, iron, and albumin. We should not forget the possible role of the hypothalamic-pituitary-adrenal axis in obese individuals [34–36]; in this study, free urinary cortisol was also decreased after BS. Our results are consistent with the demonstrated existence of active cross-talks between angiotensin II and insulin signaling, as well as a role of sub inflammation in contributing to insulin resistance in obese hypertensive subjects [37–41]. Further studies are required for a full comprehension of the interplay between RAAS and insulin sensitivity in obese individuals. The possible role of PRA in the characterization of metabolic phenotype of “remitted obese” is supported by investigations on mice lacking renin (*Ren1c*), a strain prone to diet-induced obesity. The *Ren1c*^−/−^ mice are lean, insulin sensitive, and are resistant to diet-induced obesity without changes in food intake and physical activity. In *Ren1c*^−/−^ mice enhanced energy expenditure and are resistant to development of HFD-induced obesity [42]. Instead, transgenic rodents overexpressing human renin are obese due to increased food intake and exhibit hyperglycemia, hyperinsulinemia, hyperlipidemia, and insulin resistance [43]. In contrast, the role of gender seems to be related to renin activity; female mice overexpressing human renin are protected from HFD-induced obesity. Furthermore, dysfunctional adipose tissue can partially account for the alterations of RAAS signaling influencing the surrounding organs, including the vasculature. It is well known the paracrine communication between adipose tissue and skeletal muscle, pancreas and cardiovascular system with the release of adipokines, cytokines and other small molecules, such as extracellular vesicles (EV) deputed to vehicle small RNAs (microRNAs) among others [44]. For example, miR-27a, which plays a critical for obesity by regulating insulin resistance in adipocytes, seems to facilitate the crosstalk between adipocytes and skeletal muscle, inducing insulin resistance by PPARG [45]. Bariatric surgery leads to a remission/resolution of T2D, but its precise mechanisms are not yet fully understood and the mechanisms of actions are complex, but it seems that the induction of satiety occurs by direct pressure or contact of the band with the gastric wall, or via vagal nerve signaling [46]. Beside the physiology, it is also true that a small number of studies demonstrated the role of microRNA after BS in providing a specific metabolic pattern [47–49]: about 90% down-regulation of miR-122 and a reduction of miR-342-3p, miR-320, miR-139-5p and miR-146a might regulate metabolic processes such as the citric acid cycle (TCA) cycle, glucose transport, pentose phosphate pathway, fatty-acid synthesis, mitochondrial oxidation, gluconeogenesis, and glycolysis. Obesity is recognized as a major cause of hypertension, and the combination of obesity and hypertension is recognized as a pre-eminent cause of CV risk. An additional important factor in the CV risk associated with obesity and hypertension is the role played by obesity in the development of T2D. Efforts aimed at diminishing the incidence and impact of diabetes, therefore, including both lifestyle changes and the appropriate use of antihypertensive and anti‐obesity therapies, are an essential part of the overall therapeutic plan. The recommendation to use RAAS inhibitors after coronary artery bypass grafting (CABG) in order to reduce MACE (major adverse cardiovascular events) [50] are consistent with our observation about the reliable predictive power of Renin after bariatric surgery, among a wide variety of different procedures. Finally, our results point to possible clinical implications. Selection of patients for bariatric surgery is still an open question. Determination of metabolically healthy obese subjects, for whom surgery is probably not indicated, seems possible only through genetic studies [51]. The response of individual subjects to surgery depends on the aim of surgery; for instance, prevention of mortality is more effective above a given age, while prevention of diabetes is valid for all ages [52]. Response to surgery also depends on age of patients, on initial BMI, and on the type of surgery [53, 54]. Remission of diabetes also depends on the duration of diabetes [55]. The predictive model of persistent hypertension in spite of weight loss is promising, but it should be confirmed for other surgeries, for instance malabsorptive surgeries (biliopancreatic diversion), mixed surgeries (gastric bypass), or restrictive surgeries (sleeve gastrectomy), all more effective than LAGB [54]. In the meanwhile, patients with persistent hypertension should receive close supervision and more intensive treatment to lower blood pressure, even because left ventricular hypertrophy does not regress in hypertensive patients [56].

## Conclusions

In obese subjects supine PRA is higher in hypertensive than in normotensive subjects, with a trend for progressively higher with normal glucose tolerance, impaired glucose tolerance and diabetes. Supine PRA correlates with HOMA-IR, and interestingly that both PRA and HOMA-IR correlate with ferritin. Decrease of supine PRA at 1 year after BS only occurs in hypertensive subjects, as previously reported, with no difference among groups of glucose tolerance. Decrease of supine PRA correlated with decrease of HOMA-IR and with decrease of ferritin, suggesting that changes of ferritin are mechanistically linked to change of HOMA-IR and of supine PRA. Similar, albeit less significant, was the behavior of upright PRA, and of both supine and upright aldosterone. Higher supine PRA and worse glucose tolerance (and FPG and 2hPG) predict persistence of hypertension after bariatric surgery in spite of weight loss.

## Data Availability

Data are available on request.

## Abbreviations

PRA: Plasma renin activity (supine and upright)
ALD: Aldosterone (supine and upright)
HbA1c: Haemoglobin A1c
FPG: Fasting plasma glucose
1hPG: 1h plasma glucose after OGTT
2hPG: 2h plasma glucose after OGTT
HOMA-IR: Homeostasis model assessment for insulin resistance
OGTT: Oral glucose tolerance test
BMI: Body mass index
GT: Glucose tolerance (NGT = normal; IGT = impaired; T2D = type 2 diabetes)
